# Transcriptome analysis of reproductive tissue and intrauterine developmental stages of the tsetse fly (*Glossina morsitans morsitans*)

**DOI:** 10.1186/1471-2164-11-160

**Published:** 2010-03-09

**Authors:** Geoffrey M Attardo, José MC Ribeiro, Yineng Wu, Matthew Berriman, Serap Aksoy

**Affiliations:** 1 Department of Epidemiology and Public Health, Yale University School of Medicine, New Haven, CT, USA; 2 Section of Vector Biology, Laboratory of Malaria and Vector Research, National Institute of Allergy and Infectious Diseases, National Institutes of Health, Rockville, Md, USA; 3 Wellcome Trust Sanger Institute, Hinxton, CB10 1SA, UK

## Abstract

**Background:**

Tsetse flies, vectors of African trypanosomes, undergo viviparous reproduction (the deposition of live offspring). This reproductive strategy results in a large maternal investment and the deposition of a small number of progeny during a female's lifespan. The reproductive biology of tsetse has been studied on a physiological level; however the molecular analysis of tsetse reproduction requires deeper investigation. To build a foundation from which to base molecular studies of tsetse reproduction, a cDNA library was generated from female tsetse (*Glossina morsitans morsitans*) reproductive tissues and the intrauterine developmental stages. 3438 expressed sequence tags were sequenced and analyzed.

**Results:**

Analysis of a nonredundant catalogue of 1391 contigs resulted in 520 predicted proteins. 475 of these proteins were full length. We predict that 412 of these represent cytoplasmic proteins while 57 are secreted. Comparison of these proteins with other tissue specific tsetse cDNA libraries (salivary gland, fat body/milk gland, and midgut) identified 51 that are unique to the reproductive/immature cDNA library. 11 unique proteins were homologus to uncharacterized putative proteins within the NR database suggesting the identification of novel genes associated with reproductive functions in other insects (hypothetical conserved). The analysis also yielded seven putative proteins without significant homology to sequences present in the public database (unknown genes). These proteins may represent unique functions associated with tsetse's viviparous reproductive cycle. RT-PCR analysis of hypothetical conserved and unknown contigs was performed to determine basic tissue and stage specificity of the expression of these genes.

**Conclusion:**

This paper identifies 51 putative proteins specific to a tsetse reproductive/immature EST library. 11 of these proteins correspond to hypothetical conserved genes and 7 proteins are tsetse specific.

## Background

Tsetse flies (Diptera: Glossinidae) are important agricultural and medical vectors responsible for the transmission of African trypanosomes, the agents of sleeping sickness disease in humans and nagana in animals. Human sleeping sickness has resurged in Africa necessitating the development and reevaluation of control strategies [1]. In the past, vector population control has been a successful strategy in reduction of trypanosome transmission. In particular, the success of population reduction based control strategies has resulted from the low reproductive potential of tsetse flies. The knowledge gained from molecular aspects of tsetse reproductive biology has the potential to yield new insights important for increasing the efficiency and decreasing the cost and complexity of the currently available tool set.

Tsetse has a unique reproductive physiology and developmental cycle. They undergo viviparous reproduction (intrauterine development and parturition of live offspring). Viviparous reproduction has arisen independently in other flies and in other orders of insects [2]. This process is specialized in tsetse. In tsetse the entire larval developmental cycle is intrauterine and the mother supplies nutrients to her offspring in the form of a milk secretion from a specialized gland (milk gland) [3]. Female flies develop a single larva at a time. As a result a single female has the capacity to generate ~8 offspring in her lifecycle. This is significantly less than most other Diptera, such as mosquitoes, that are capable of generating hundreds of offspring in the span of a single life cycle. The low reproductive rate in tsetse represents a potential target for vector control as disruption of this process could have dramatic effects on population density.

Previous gene discovery projects for tsetse have focused on the adult fat body/milk gland [4], midgut [5] and salivary gland [6] organs in the tsetse species *Glossina morsitans morsitans*. Multiple genes with potential importance for the reproductive cycle were identified in the fat body/milk gland library analysis. In particular four cDNAs have been characterized in more detail and identified as proteins synthesized by the milk gland for larval nourishment. These proteins include the major milk protein (*gmmmgp1*) [7], transferrin (*gmmtsf*) [8], milk gland protein 2 (*gmmmgp2*) and milk gland protein 3 (*gmmmgp3*) [9].

In order to perform an in depth study of reproduction associated genes, a new cDNA library was generated with a pool of mRNA from reproductive tissues (uterus, ovaries and spermathica) and intrauterine larvae at all stages of development (embryo 1^st^, 2^nd ^and 3^rd ^instar larvae). cDNAs from this library were randomly sequenced and resulting sequences were manually annotated. Many of the annotated sequences were orthologous to genes associated with oogenesis, embryogenesis and larvigenesis in other insects. Comparison of this library with other tsetse tissue specific libraries was informative in the identification of reproductive specific transcripts with uncharacterized orthologs in other insects, as well as transcripts of putative genes that show no homology to any genes in the NCBI non-redundant database.

## Results and discussion

### General description of the reproductive/immature cDNA library

A total of 3438 ESTs were sequenced from the reproductive library of which 3048 were used for contig generation after quality control screening. Following assembly, the total number of unique contigs derived from the ESTs was 1391. Average length of the contigs was 1125 bp with a maximum of 5302 bp and a minimum of 88 bp.

The contigs from this library were combined with EST sequence data from the other tissue specific tsetse cDNA libraries. The combined library consists of 16509 contigs derived from published cDNA libraries including adult fat body/milk gland [4], midgut [5], salivary gland (in press) and the reproductive/immature ESTs described here. After assembly, reproductive/immature library specific contigs were identified and annotated.

**Figure 1:**
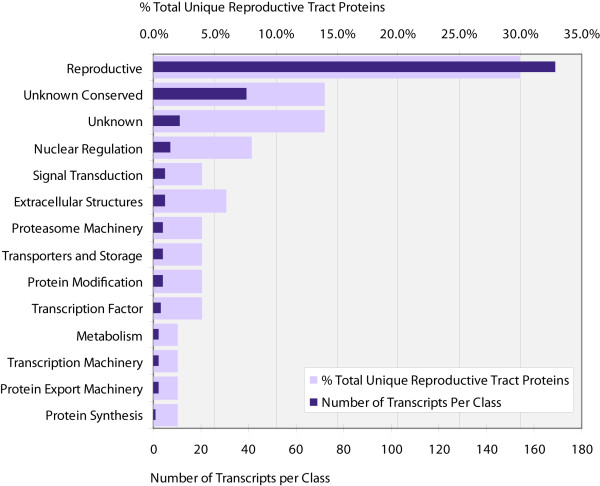
**Statistics on all predicted reproductive/immature proteins**. This graph represents all putative proteins within the reproductive/immature cDNA library. Light bars represent the percentage of predicted proteins categorized by function from the reproductive/immature library. Dark bars represent the number of ESTs associated with the genes within each category.

Analysis and categorization of proteins extrapolated from reproductive/immature EST sequences was performed (Figure 1). There were a total of 520 unique putative proteins identified in the reproductive/immature cDNA library; 475 transcripts represented full-length cDNAs, 41 were truncated and 4 were fragments. Identification of signal peptides and anchoring motifs associated with the putative proteins predicted that 412 are cytoplasmic proteins, 57 have signal peptides suggesting secretion and 18 are anchored to the cell membrane. Results for the 33 remaining sequences were difficult to interpret due to the fact that they were either fragments or the results were ambiguous.

The profile of this library shows that hypothetical conserved proteins constitute 13% of the entire library, followed by proteins associated with metabolism and protein synthesis at 10% and 11%, respectively. The categories that had the highest number of transcripts differed from the ones that had the highest number of actual genes. Factors associated with protein synthesis were the most highly expressed in the library with 416 transcripts, followed by cytoskeletal genes at 206 transcripts and then reproductive proteins at 188 transcripts. These results are logical as reproductive tissues and immature stages synthesize a large amount of protein for oogenesis, embryogenesis and nutrient storage. High levels of expression of cytoskeletal genes such as *tubulin *and *actin *are required for the cellular changes undergone during oogenesis, embryogenesis and larvigenesis. Genes classified as reproductive encode proteins associated with processes such as vitellogenesis, embryonic development and larvigenesis. The reproductive classification was determined by homology to previously identified tsetse genes or to orthologs associated with these processes in other insects.

**Figure 2:**
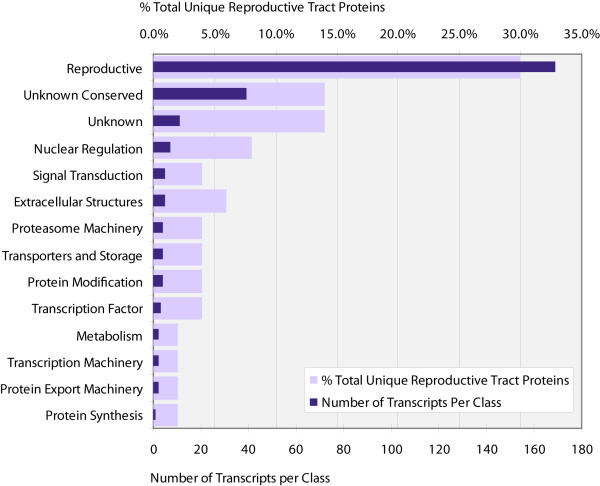
**Statistics on predicted proteins unique to the reproductive/immature library**. This graph represents putative proteins unique to the reproductive/immature cDNA library relative to fat body, midgut and salivary gland specific cDNA libraries. Light bars represent the percentage of library specific predicted proteins categorized by function. Dark bars represent the number of ESTs associated with the genes within each category.

Predicted proteins unique to the Reproductive/immature library were categorized and EST abundance for the categories was calculated (Figure 2). After filtration of nonspecific sequences, 51 library specific predicted proteins remained. Within this subgroup, reproduction associated genes contributed to 33% of the ESTs. This group of genes also had the highest number of transcripts among reproductive tract/immature exclusive genes with 169 transcripts. The second and third most abundant EST categories were hypothetical conserved (11) and unknown genes (7) with the second and third highest number of transcripts, 51 and 17, respectively.

The hypothetical conserved genes are of interest, as these genes are likely associated with reproduction in not only tsetse but in other insects as well. Characterization of these genes could provide new insights into processes associated with reproduction and development in insects. There were seven unknown genes, which returned no significant homologues from the NCBI database and thus appear to be unique to tsetse. Analysis of these genes and their products has the potential to reveal novel functions and processes associated with viviparous reproduction. These gene products may constitute potential targets for population control strategies. As these genes may be unique to tsetse, they may pose very specific targets for control mechanisms interrupting the viviparous reproductive cycle of tsetse.

### Protein orthologs expressed exclusively in the reproductive/immature library: Table 1 (Additional file 1)

With the exception of cDNAs encoding unknown proteins, the rest of the library was orthologous to putative proteins identfied in the NCBI non-redundant database. The majority of the genes were most closely related to orthologs from *Drosophila *species (81%). This is not unexpected as *Drosophila *is well characterized and is closely related to tsetse (both are on the suborder Brachycera/Muscomorpha) relative to other flies such as mosquitoes (which are Nematocera) [10]. We focused our analysis on tsetse cDNAs expressed exclusively in this library to identify putative proteins associated with tsetse's unique reproductive and developmental biology.

#### Nuclear regulation: Reproductive specific histones

We identified two putative histones limited to the reproductive library. Histone composition of nucleosomes has been shown to change in different tissues and at different developmental stages in *Drosophila *[11]. One of the histones (EZ421930) appears to be homologous to Histone H1. This particular orthologue of H1 could be associated with regulation of genomic DNA structure during specific developmental events in the developing oocyte or the developing embryo.

The second histone is homologous to histone H3 (EZ421945). A reproduction specific H3 variant (H3.3) appears to play a role in oocyte and embryonic development in mice. This variant is a maternal factor provided to the oocyte and is localized to active regions of the genome. It is thought to be associated with epigenetic reprogramming [12].

#### Protein export: gamma-SNAP

An important process that occurs during oogenesis in insects is vitellogenesis, the secretion of yolk proteins from either fat body cells or ovarian follicle cells and the uptake of those yolk proteins into the oocyte for use as raw materials during embryonic development. This process requires the transport of protein(s) across the membrane of the cell producing the protein (in the case of tsetse the ovarian follicle cells) and the membrane of the developing oocyte. A reproductive specific protein homologus to the SNAP family of proteins was identified in the library (EZ421968). In vertebrates, the SNAP proteins are required for formation of the SNARE complex which promotes the fusion of membrane lipid bilayers [13-15]. As the primary function of this protein is to mediate exocytosis and is specific to this library, it is a logical assumption that it may be a component in the machinery necessary for vitellogenesis. Further study of this gene could yield insights into the mechanisms yolk protein secretion and vitellogenesis.

#### Lipid metabolism/hormone synthesis: 17-beta-hydroxysteroid dehydrogenase type 3

Two of the library exclusive cDNAs identified (EZ421953 and EZ421957) appear to be orthologues of a 17 beta-hydroxysteroid dehydrogenase in *Drosophila melanogaster*. These enzymes are associated with the biosynthesis and inactivation of steroid hormones [16]. Although the two cDNAs code for similar proteins, they appear to represent unique genes. Steroid hormones are associated with regulation of egg development in other insects. The role of steroid hormone function in tsetse reproduction is unknown. Analysis of these genes is a potential starting point for this area of research.

#### Yolk proteins

Vitellogenesis is a major process associated with egg development. It is the synthesis and deposition of yolk proteins into a developing oocyte. Characterization of vitellogenesis was previously performed in *Glossina *[7], where a yolk protein gene identified from a fat body/milk gland tissue library was characterized and found to be predominantly expressed in the reproductive tract. In nematoceran flies such as mosquitoes, yolk proteins are generated by the fat body. In contrast, Cyclorrhaphan flies such as *D*. *melanogaster *and *Sarcophaga bullata *express their yolk proteins from both the fat body and the follicle cells of the ovary. In *Glossina *yolk proteins appear to be predominantly expressed in the ovary and are likely expressed from the follicle cells. This particular gene is one of a few that has a large number of representative ESTs present in this library (16) relative to other library specific genes. This observation supports our previous characterization as well as observations by [17] that *GmmYP *is an ovary specific gene and is the only yolk protein identified in *Glossina*. Multiple yolk proteins have been identified and compared in *D. melanogaster*, *Musca domestica *and *Sarcophaga bullata *[18-20].

#### Gonadal proteases

**Figure 3:**
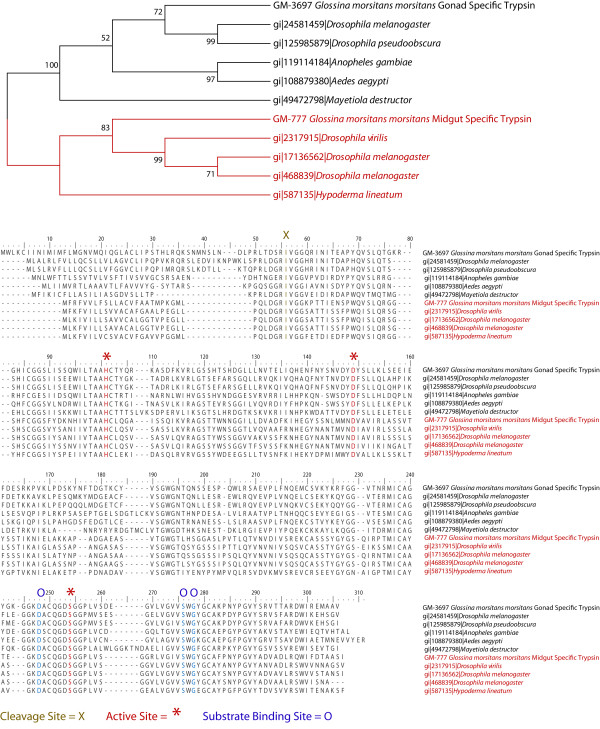
**Phylogenetic and alignment based analysis and comparison of a gonad specific trypsin (EZ421932) with a midgut specific trypsin (EU589384)**. Orthologus sequences were identified by PSI-BLAST analysis. Sequences were then aligned with standalone ClustalX followed by manual adjustments. Phylogenetic tree construction and bootstrap analysis were performed with MEGA 3.1.

Other important constituents of oocyte yolk are proteases required for the metabolism of yolk proteins and activation of other proteases during embryogenesis. One of the library specific genes appears to be homologous to a trypsin type protease (EZ421932). This trypsin could either be a component of the yolk or a larval midgut specific trypsin as the library does contain transcripts from immature stages. Other trypsin like transcripts were also identified (EZ421964 and EZ421960), however these transcripts were truncated making it difficult to perform a complete analysis. Phylogenetic comparison of trypsin EZ421932 with a previously identified midgut specific trypsin from *Glossina *suggests that these genes developed independently from each other (Figure 3a). Orthologs of the library specific trypsin were identified in other fly species. None of the homologues had been previously characterized. Alignment of this trypsin with its homologues and with midgut specific trypsins reveals structural conservation of key residues associated with the trypsin active site, substrate binding site and proteolytic cleavage site (Figure 3b). The two protein clades differ significantly in the N-terminal region of the protein. This region may be associated with secretion and/or activation of these trypsins.

#### Chorion proteins

Two putative chorion proteins (EZ421949 and EZ421934) were identified as library specific and are orthologus to chorion proteins from *Drosophila*. The protein encoded by EZ421949 is closely related to chorion protein 38 and is likely a component of the endochorion as it undergoes transcriptional activation in *Drosophila *oocytes late in oocyte development [21].

#### RNA localization

Correct localization of nurse cell generated maternal RNA within the developing oocyte is required for dorsal/ventral and anterior/posterior patterning and positioning of primordial germ cells in the developing embryo. A cDNA that is strongly orthologous to the *Drosophila *gene *tsunagi *was identified in the database (EZ421943). Tsunagi is a RNA binding protein that forms a complex with another protein called Mago Nashi. In *Drosophila *Tsunagi knockout phenotypes show failure of the oocyte nucleus to migrate, the maternal mRNA *oskar *does not localize to the posterior pole and dorsal/ventral patterning abnormalities occur [22].

#### Apterous

There are many transcription factors associated with embryonic development in insects. The ortholog for one of these factors (encoded by a gene called *apterous *(*ap*) in *Drosophila*) was identified as a library specific transcript (EZ421958). The effects of the knockout of this gene in *Drosophila *are an absence of wing and haltere development, absence of development of a group of embryonic muscles and juvenile hormone deficiency due to defects in secretory cells in the corpora allata. Indirect effects due to the absence of juvenile hormone include female sterility due to arrest of oogenesis, abnormal larval fat body breakdown, aberrant sexual behavior and premature death in adults [23].

Study of this factor and its effects on the development of the tsetse endocrine system (specifically the corpora allata) could yield important information on how juvenile hormone regulates oogenesis and pregnancy in tsetse.

#### Doublecortin

An essential factor for the development of the embryonic brain and nervous system is a protein called Doublecortin (*dcx*). Mutations in this gene in mammalian systems result in disruption of cortical neuronal migration [24]. An orthologous cDNA to *dcx *was identified as a library specific gene. A thorough investigation of how this gene functions in invertebrates has yet to be performed.

#### Cuticular proteins

A group of proteins associated with larval development that were exclusive to this library were cuticular proteins. Three putative proteins were identified (EZ421946, EZ421965 and EZ421951), two of which (EZ421946 and EZ421951) are orthologous to ecdysone dependant pupal cuticle proteins. Previous work has been performed on tsetse cuticle proteins expressed during the different developmental stages [25]. It was observed that a large number of proteins are synthesized by the late third instar larvae relative to first and second instar larvae. This is logical as the intrauterine environment is hydrated and protective thereby not requiring the larva to protect itself from a hostile environment. The burst of expression of third instar cuticle proteins could be associated with larvae that are preparing for the stresses of parturition, wandering and pupation.

#### Fat body protein 2

Another transcript identified in the analysis of the library is an ortholog to the *fat body Protein 2 (FBP2) *gene (EZ421940) which is homologous to an alcohol dehydrogenase protein (ADH). This protein is thought to assist in the degradation of the fat body during metamorphosis [26].

#### Hexamerins

**Figure 4:**
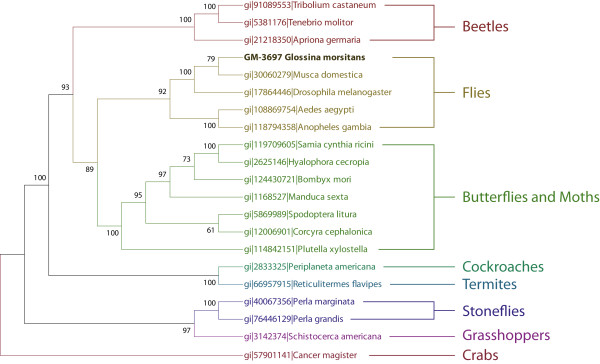
**Phylogenetic and alignment based analysis and comparison of a library specific hexamerin protein (EZ421932)**. Orthologus sequences were identified by PSI-BLAST analysis. Sequences were then aligned with standalone ClustalX followed by manual adjustments. Phylogenetic tree construction and bootstrap analysis were performed with MEGA 3.1.

Hexamerins (Arylphorins) are larva specific storage proteins. Two hexamerin/arylphorin type proteins were identified as library specific (EZ421969/EZ421959 and EZ421955). These cDNAs were very abundant relative to other unique genes with representative EST abundance of 23 and 4 transcripts respectively. Phylogenetic analysis of EZ421969/EZ421959 was performed with hexamerins from other insect species and a hemocyanin from crabs as an outgroup (Figure 4). Tsetse hexamerin forms a group with the other cyclorrhaphan flies (*Drosophila *and *Musca domestica*) and then on a larger scale with the nematoceran diptera (mosquitoes). The next closest group is the Lepidoptera (butterflies and moths) followed by the Coleoptera (beetles). This is followed by the more primitive hemimetabolus insects, Plecoptera (stoneflies), Dictyoptera (cockroaches and termites) and Orthoptera (grasshoppers). The relationship between these proteins follows that predicted by current morphological and molecular systematic analysis of insects at the "Tree of Life "http://tolweb.org/tree/" [27]. Phylogenetic analysis of these genes shows them to be informative from a taxonomic viewpoint as there are many hexamerin proteins present in the NCBI database. They are also of biological interest as they are developmentally and tissue specifically regulated [28] and are essential for the transition from immature to the adult stages of development.

### Tissue and developmental specificity analysis of unknown and hypothetical conserved genes: Table 2 (Additional file 2)

The search for library specific sequences revealed 18 hypothetical conserved and unknown genes. Of these genes 11 hypothetical conserved and 7 unknown gene sequences were identified. To verify the specificity of these transcripts RT-PCR analysis was performed using cDNAs from pupa, larva, reproductive tract (ovaries, uterus, spermathica) and the remaining carcass mRNA from 10 adult flies at varying ages (Table 2). The cDNAs used in this assay were generated from tissue samples independent of the ones used to generate the library. None of the genes were expressed in the carcass, confirming their library specific nature. A total of seven genes, five hypothetical conserved (EZ421939, EZ421950, EZ421936, EZ421937 and EZ421938) and two unknowns (EZ421944 and EZ421929) were found to be exclusive to the reproductive tract. These proteins could be associated with functions in adult tissues such as the ovaries (oogenesis), uterus or spermatheca. Identification of signal peptides and transmembrane domains in these proteins offer clues for their localization and function. EZ421939 and EZ421950 both have predicted secretion signals suggesting that these proteins are secreted from the reproductive tract. Neither EZ421936 nor EZ421937 have signal peptides; however they both have one transmembrane domain, with 76% of the protein predicted to be extracellular.

Three hypothetical conserved genes (EZ421923, EZ421935 and EZ421956) were identified as larva specific. All three putative proteins contain signal peptides suggesting secretion. A hypothetical conserved gene was found to be pupa specific (EZ421925) and has a predicted signal peptide and two transmembrane domains with 50% of the protein predicted to be extracellular. EZ421954 was detectable in both reproductive tract and larva and has a secretion signal.

Finally, three unknown genes were expressed in both larva and pupa specific cDNAs (EZ421961, EZ422129 and EZ421927). The localization of EZ421961 and EZ422129 could not be determined as they are partial cDNAs.

Hypothetical and unknown genes are of particular interest as they encode proteins either present in other systems with yet unknown functions, or proteins completely novel to the tsetse system. The evolutionary biology of the unknown genes is of particular interest. Determination of the origins and functions of these proteins will be important to understanding the evolutionary process by which tsetse developed viviparous reproduction. This line of research can also yield the identification of tsetse specific target genes, which could be blocked to disrupt reproduction with minimal environmental effects.

## Conclusions

Determining tsetse's transcriptome from the reproductive/immature cDNA library has yielded new information on putative proteins expressed in the reproductive tract and immature intrauterine developmental stages of tsetse. Information associated with similar proteins characterized in other organisms can now be placed into the context of tsetse's reproductive and developmental biology to elucidate its reproductive physiology. This will be important to understand how these genes have been adapted to perform in a viviparous system. Also of importance is the identification of genes orthologous in other organisms that remain uncharacterized, as well as the identification of genes entirely novel to tsetse. Analysis and characterization of these genes will reveal information fundamental to insect reproduction and development. Given the low reproductive capacity of tsetse, molecular data on reproduction specific processes has the potential to reveal novel mechanisms which could be exploited to control tsetse population levels and trypanosomiasis transmission.

## Methods

### Tsetse

The *Glossina morsitans morsitans *colony maintained in the insectary at Yale University was originally established from puparia from fly populations in Zimbabwe. Flies are maintained at 24 ± 1°C with 50-55% relative humidity, and receive defibrinated bovine blood every 48 h using an artificial membrane system [29].

### Tissue collection and RNA extraction

Reproductive tissues and immature developmental stages were dissected from female flies at various stages of pregnancy. Dissections were performed in PBS and tissues were snap frozen in liquid nitrogen and stored at -80°C. Reproductive tissues included ovaries, uterus and spermathica. Immature stages included embryo, first, second and third instar larvae. Total RNA was isolated from samples using TRIzol^®^Reagent (Invitrogen, Carlsbad, CA) according to manufacturer's instructions.

### cDNA library construction, sequencing, EST clustering and data analysis

The library was commercially constructed in Express 1 vector from 1 mg of total RNA derived from reproductive tissues and immature developmental stages (OPEN biosystems, Huntsville, AL). Each clone was sequenced using a T3 or T7 primer using ABI Big Dye terminator kits on an ABI 3730 sequencing machine. ESTs were trimmed of primer and vector sequences, clustered and compared with other databases as has been described previously [30]. The entire database can be downloaded from the Aksoy lab website http://aksoylab.yale.edu/Links.html.

Functional annotation of the transcripts were performed using BLASTX to compare nucleotide sequences to the NR protein database at NCBI [31] as well as to KOG [32] and GO [33] databases. Detection of conserved protein domains was performed using rpsBLAST [34] and Pfam [35]. The predicted protein translations were submitted to the SignalP server to help identify those products that could be secreted [36]. We compared these transcripts to several proteomes obtained from Flybase [37] (*D. melanogaster*) and ENSEMBL [38] (*An. gambiae*).

A subsequent tsetse EST assembly of the combined EST data dated 12/17/2008 has been produced by the International *Glossina *Genomics Initiative (IGGI) consortium and is available at http://www.genedb.org. The contig sequences in this paper reference the identifiers assigned in the initial clustering. The tables included in this manuscript and supplementary spreadsheets include the analogous GeneDB identifiers and hyperlinks to the appropriate data pages for each sequence.

### Phylogenetic analysis

Phylogenetic analysis of the library specific hexamerin and trypsin genes was performed via multiple steps. Putative orthologues were identified by PSI-BLAST [34] search of the NCBI NR database and compiled within a FASTA file. Sequences were aligned using standalone ClustalX software [39]. Alignments were hand edited in BioEdit [40]. Incomplete and poorly aligned sequences were removed. Phylogenetic tree construction and bootstrap analysis were performed in MEGA 3.1 [41].

### RT-PCR expression analysis

cDNA pools used for RT-PCR analysis were generated from total RNA isolated from reproductive tract (uterus, spermathica and ovaries), 1^st ^- 3^rd ^instar larvae, pupa and the remaining carcass after removal of reproductive tract and intrauterine offspring. Total RNA was isolated from samples using TRIzol^® ^Reagent (Invitrogen, Carlsbad, CA) according to manufacturer's instructions. One μg of total RNA from each sample was used to synthesize each pool of cDNA using the Superscript Double Stranded cDNA Synthesis kit (Invitrogen). Tubulin levels were used to determine the dilution factor and final amount of cDNA from each pool used in the PCR reactions. After equilibration with tubulin, PCR analysis was performed with primers specific to each of the hypothetical conserved and unknown proteins identified within the library. Primer sequences used for the RT-PCR analysis are listed in Additional File 3. PCR conditions were as follows: 95°C for 3 minutes (1×), 95°C for 30 secs, 60°C for 45 secs, 72°C for 1 min (25×) and 72°C for 5 min (1×). PCR products were run on a 1% agarose gel and stained with ethidium bromide. Presence or absence of transcript was determined by visual examination of gel staining. Primer sequences are included in the primer list supplementary file.

## Abbreviations

EST: expressed sequence tag; kbase: kilobase; NR: nonredundant; nt: nucleotide; RT-PCR: reverse transcriptase polymerase chain reaction; cDNA: copyDNA.

## Authors' contributions

GA prepared the biological material for library construction, supervised tissue expression experiments, performed data analysis and prepared the manuscript. JMCR performed data analysis and contributed to the manuscript. YW performed tissue specific gene expression experiments. MB participated in sequencing the cDNA library. SA participated in library construction, data analysis and contributed to the manuscript. All authors have read and approved the final manuscript.

## Supplementary Material

Additional file 1 **Table 1**. This file contains the list of predicted (reproductive/immature library specific and non specific) reproduction associated proteins. Click here for file

Additional file 2 **Table 2**. This file contains the list of reproductive/immature library specific hypothetical conserved and unknown proteins with RT-PCR expression data. Click here for file

Additional file 3 **Primer list supplement**. This file contains a table of the primers used in the tissue specificity RT-PCR analysis from table 2. Click here for file
