# Supplementary material for: Transcriptome analysis of reproductive tissue and intrauterine developmental stages of the tsetse fly (Glossina morsitans morsitans)
Source: BMC Genomics. 2010 Mar 9;11:160. doi: 10.1186/1471-2164-11-160 (PMC2846916; doi:10.1186/1471-2164-11-160)
Supplement: Additional file 1 — Table 1. This file contains the list of predicted (reproductive/immature library specific and non specific) reproduction associated proteins. [file 1471-2164-11-160-S1.DOC]

**Table 1: Predicted library specific and non specific reproductive associated proteins.**

| **Database ID (Accession #)** | **GeneDB Identifier** | **# of Reproductive ESTs** | **Total # of ESTs** | **Status** | **Description for fasta file** | **Best match to NR protein database** | **E value** | **Species of Closest Homologue** |
| --- | --- | --- | --- | --- | --- | --- | --- | --- |
| **Library specific reproductive associated proteins** | | | | | | | | |
| **Nuclear Regulation** | | | | | | | | |
| GM-4057  (EZ421933) | [cn9250](http://www.genedb.org/genedb/Search?name=cn9250&organism=glossina) | 2 | 2 | Full | Predicted ATPase, nucleotide-binding | XP_001848618 | 6E-092 | Culex quinquefasciatus |
| GM-9036  (EZ421962) | [FN182316](http://www.ncbi.nlm.nih.gov/nucest/224506519?rid=dc3wt6fe01s&blast_rank=1&dopt=genbank&log$=nucltop)* | 2 | 2 | Truncated | Borealin-related - truncated | XP_002051111 | 4E-055 | Drosophila virilis |
| GM-9043  (EZ421945) | [cn8927](http://www.genedb.org/genedb/Search?name=cn8927&organism=glossina) | 2 | 2 | Full | Similar to H3 histone, family 2 isoform 2 | XP_002422654 | 2E-069 | Pediculus humanus corporis |
| GM-17645  (EZ421930) | [cn13228](http://www.genedb.org/genedb/Search?name=cn13228&organism=glossina) | 1 | 1 | Full | Histone H1 | XP_002052264 | 3E-070 | Drosophila virilis |
| **Transcriptional Machinery** | | | | | | | | |
| GM-17309  (EZ421924) | [cn8844](http://www.genedb.org/genedb/Search?name=cn8844&organism=glossina) | 1 | 1 | Full | Transcription factor CBF, beta subunit - truncated | XP_002046369 | 1E-111 | Drosophila virilis |
| GM-9032  (EZ421941) | [cn8835](http://www.genedb.org/genedb/Search?name=cn8835&organism=glossina) | 2 | 2 | Full | Gonadal Ca2+-binding protein Regucalcin/SMP30 | BAA99282 | 1E-148 | Sarcophaga peregrina |
| GM-9073  (EZ421967) | [cn9309](http://www.genedb.org/genedb/Search?name=cn9309&organism=glossina) | 2 | 2 | Truncated | Chorion factor CF2 - truncated | XP_001356732 | 3E-019 | Drosophila pseudoobscura |
| GM-9070  (EZ421966) | [cn9289](http://www.genedb.org/genedb/Search?name=cn9289&organism=glossina) | 2 | 2 | Truncated | Putative zinc transporter - truncated | XP_001957884 | 1E-128 | Drosophila ananassae |
| **Lipid Metabolism/Hormone Synthesis** | | | | | | | | |
| GM-9074  (EZ421953) | [cn9304](http://www.genedb.org/genedb/Search?name=cn9304&organism=glossina) | 2 | 2 | Full | 17 Beta-hydroxysteroid dehydrogenase type 3, HSD17B3 | XP_002002062 | 3E-087 | Drosophila mojavensis |
| GM-9044  (EZ421957) | [cn8912](http://www.genedb.org/genedb/Search?name=cn8912&organism=glossina) | 2 | 2 | Truncated | 17 Beta-hydroxysteroid dehydrogenase type 3 | XP_002002062 | 6E-077 | Drosophila mojavensis |
| **Protein Export** | | | | | | | | |
| GM-9083  (EZ421968) | [cn8190](http://www.genedb.org/genedb/Search?name=cn8190&organism=glossina) | 2 | 2 | Truncated | Protein required for fusion of vesicles in vesicular transport, gamma-SNAP - truncated | XP_315648 | 1E-117 | Anopheles gambiae str. PEST |
| **Protein Modification** | | | | | | | | |
| GM-9037  (EZ421963) | [cn8838](http://www.genedb.org/genedb/Search?name=cn8838&organism=glossina) | 2 | 2 | Truncated | Ubiquitin C-terminal hydrolase - truncated | XP_002087630 | 2E-077 | Drosophila yakuba |
| GM-9053  (EZ421948) | [cn9088](http://www.genedb.org/genedb/Search?name=cn9088&organism=glossina) | 2 | 2 | Full | SCF ubiquitin ligase, Skp1 component | XP_001987879 | 5E-049 | Drosophila grimshawi |
| GM-9071  (EZ421952) | [cn9291](http://www.genedb.org/genedb/Search?name=cn9291&organism=glossina) | 2 | 2 | Full | Ethanolamine-P-transferase GPI11/PIG-F | XP_002008466 | 1E-103 | Drosophila mojavensis |
| **Protein Synthesis** | | | | | | | | |
| GM-17566  (EZ421926) | [LAR-005O06.g](http://www.genedb.org/genedb/Search?name=LAR-005O06.g&organism=glossina) | 1 | 1 | Full | 60S ribosomal protein L31 | XP_002049279 | 1E-061 | Drosophila virilis |
| **Sperm Function** | | | | | | | | |
| GM-356  (EZ421931) | [cn14492](http://www.genedb.org/genedb/Search?name=cn14492&organism=glossina) | 99 | 99 | Full | Sperm mitochondria-associated cysteine-rich protein | XP_001891729 | 5E-032 | Brugia malayi |
| **Oogenesis** | | | | | | | | |
| GM-1486  (AY490789) | [cn590](http://www.genedb.org/genedb/Search?name=cn590&organism=glossina) | 16 | 23 | Full | Yolk Protein | AAR84615 | 0.0 | Glossina morsitans morsitans |
| GM-9038  (EZ421942) | [cn13641](http://www.genedb.org/genedb/Search?name=cn13641&organism=glossina) | 2 | 2 | Full | Receptor targeting protein Lin-7 | XP_001998460 | 1E-100 | Drosophila mojavensis |
| GM-6932  (EZ421970) | [FN184566](http://www.ncbi.nlm.nih.gov/nucest/224508154?rid=dc6u163701n&blast_rank=1&dopt=genbank&log$=nucltop)* | 3 | 3 | Fragment | Choriogenin H - fragment | NP_001098277 | 2E-004 | Oryzias latipes |
| GM-3697  (EZ421932) | [cn8949](http://www.genedb.org/genedb/Search?name=cn8949&organism=glossina) | 9 | 9 | Full | Gonadal trypsin | XP_001968573 | 2E-093 | Drosophila erecta |
| GM-9040  (EZ421964) | [cn8867](http://www.genedb.org/genedb/Search?name=cn8867&organism=glossina) | 2 | 2 | Truncated | Trypsin-like serine protease precursor - truncated | AAF91346 | 4E-095 | Glossina morsitans morsitans |
| GM-5921  (EZ421960) | [cn9053](http://www.genedb.org/genedb/Search?name=cn9053&organism=glossina) | 4 | 4 | Truncated | Trypsin expressed in reproductive organs - truncated | XP_002006762 | 8E-069 | Drosophila mojavensis |
| GM-4318  (EZ421934) | [cn8932](http://www.genedb.org/genedb/Search?name=cn8932&organism=glossina) | 7 | 7 | Full | Chorion protein | XP_002100538 | 2E-037 | Drosophila yakuba |
| GM-9055  (EZ421949) | [cn9338](http://www.genedb.org/genedb/Search?name=cn9338&organism=glossina) | 2 | 2 | Full | Chorion protein | XP_002101136 | 2E-050 | Drosophila yakuba |
| GM-9041  (EZ421943) | [cn8854](http://www.genedb.org/genedb/Search?name=cn8854&organism=glossina) | 2 | 2 | Full | U1 small nuclear ribonucleoprotein (RRM superfamily) | XP_002004225 | 6E-084 | Drosophila mojavensis |
| **Embryonic Development** | | | | | | | | |
| GM-9061  (EZ421958) | [cn9221](http://www.genedb.org/genedb/Search?name=cn9221&organism=glossina) | 2 | 2 | Truncated | Protein apterous homolog - dorsal ventral embryonic axis development | XP_002061119 | 2E-055 | Drosophila willistoni |
| GM-9059  (EZ421972) | [cn9079](http://www.genedb.org/genedb/Search?name=cn9079&organism=glossina) | 2 | 2 | Fragment | Neuronal migration protein doublecortin (Lissencephalin-X) (Lis-X) (Doublin) - truncated | XP_001972764 | 2E-067 | Drosophila erecta |
| **Larval Development** | | | | | | | | |
| GM-9051  (EZ421946) | [GMre-18f02.q1k](http://www.genedb.org/genedb/Search?name=GMre-18f02.q1k&organism=glossina) | 1 | 1 | Full | Pupal cuticle protein Edg-78E precursor | XP_002047163 | 2E-044 | Drosophila virilis |
| GM-9065  (EZ421965) | [cn9243](http://www.genedb.org/genedb/Search?name=cn9243&organism=glossina) | 2 | 2 | Truncated | Cuticle protein - truncated | XP_002093666 | 6E-060 | Drosophila yakuba |
| GM-9067  (EZ421951) | [FN184343](http://www.ncbi.nlm.nih.gov/nucest/224509429?rid=dc758c4c01n&blast_rank=1&dopt=genbank&log$=nucltop)* | 2 | 2 | Full | Pupal cuticle protein Edg-91 precursor (Ecdysone-dependent protein 91) | XP_002054703 | 3E-012 | Drosophila virilis |
| GM-6934  (EZ421940) | [cn14076](http://www.genedb.org/genedb/Search?name=cn14076&organism=glossina) | 3 | 3 | Full | 15-Hydroxyprostaglandin dehydrogenase or related dehydrogenase | CAA26412 | 6E-097 | Sarcophaga peregrina |
| GM-2235/GM-4700  (EZ421959/EZ421969) | [cn9193](http://www.genedb.org/genedb/Search?name=cn9193&organism=glossina) | 17 | 17 | Truncated | Hexamerin LSP-2 - truncated | AAC24157 | 0.0 | Calliphora vicina |
| GM-5919  (EZ421955) | [cn9579](http://www.genedb.org/genedb/Search?name=cn9579&organism=glossina) | 4 | 4 | Truncated | Arylphorin - truncated | XP_002003706 | 1E-164 | Drosophila mojavensis |
| **Non-specific reproductive associated proteins** | | | | | | | | |
| **Extracellular Structure** | | | | | | | | |
| GM-2454  (EZ422105) | [cn3205](http://www.genedb.org/genedb/Search?name=cn3205&organism=glossina) | 8 | 15 | Truncated | Chitinase - truncated | XP_002003706 | 0.0 | Drosophila virilis |
| **Embryonic Development** | | | | | | | | |
| GM-7375  (EZ422045) | [cn9220](http://www.genedb.org/genedb/Search?name=cn9220&organism=glossina) | 1 | 2 | Truncated | Polycomb protein esc (Protein extra sex combs) extra sex combs - truncated | O16023 | 0.0 | Musca domestica |
| GM-4419  (EZ423586) | [cn9059](http://www.genedb.org/genedb/Search?name=cn9059&organism=glossina) | 2 | 4 | Full | Female-specific protein transformer | ACS34687 | 7E-044 | Lucilia cuprina |
| GM-8841  (EZ422193) | [cn9687](http://www.genedb.org/genedb/Search?name=cn9687&organism=glossina) | 1 | 2 | Truncated | Spondin, extracellular matrix protein - truncated | XP_002006775 | 8E-068 | Drosophila mojavensis |
| GM-8842  (EZ422049) | [cn10265](http://www.genedb.org/genedb/Search?name=cn10265&organism=glossina) | 1 | 2 | Truncated | Spondin, extracellular matrix protein - truncated | XP_002006775 | 4E-069 | Drosophila mojavensis |
| **Larval Development** | | | | | | | | |
| GM-711  (AF368908) | [cn268](http://www.genedb.org/genedb/Search?name=cn268&organism=glossina) | 8 | 45 | Full | Transferrin | AAM46784 | 0.0 | Glossina morsitans |
| GM-999  (DQ294227) | [cn2986](http://www.genedb.org/genedb/Search?name=cn2986&organism=glossina) | 0 | 33 | Full | Fat body lipocalin / Milk Gland Protein | ABC48943 | 1E-111 | Glossina morsitans |

* Starred identifiers represent links to the NCBI dbEST database as analogus sequences are not found at GeneDB (<http://www.ncbi.nlm.nih.gov/dbEST/>) [42].
