# Supplementary material for: Transcriptome analysis of reproductive tissue and intrauterine developmental stages of the tsetse fly (Glossina morsitans morsitans)
Source: BMC Genomics. 2010 Mar 9;11:160. doi: 10.1186/1471-2164-11-160 (PMC2846916; doi:10.1186/1471-2164-11-160)
Supplement: Additional file 2 — Table 2. This file contains the list of reproductive/immature library specific hypothetical conserved and unknown proteins with RT-PCR expression data. [file 1471-2164-11-160-S2.DOC]

**Table 2: Reproductive/immature library specific hypothetical conserved and unknown proteins with expression data.**

| **Database ID**  **(Accession #)** | **GeneDB Identifier** | **# of ESTs** | **Status** | **SigP Result** | **Trans membrane Domain** | **Best match to NR protein database** | **E- Value** | **Match** | **Species of best match** | **RT-PCR Tissue Specificity Data** | | | |
| --- | --- | --- | --- | --- | --- | --- | --- | --- | --- | --- | --- | --- | --- |
| **Hypothetical Conserved** | | | | | | | | | | Carcass | Reproductive Tract | Larva | Pupa |
| GM-6933  (EZ421939) | [cn9272](http://www.genedb.org/genedb/Search?name=cn9272&organism=glossina) | 3 | Full | Signal Peptide | No | GG15234 | 5E-064 | XP_001971910 | Drosophila erecta |  | **X** |  |  |
| GM-9063  (EZ421950) | [cn9253](http://www.genedb.org/genedb/Search?name=cn9253&organism=glossina) | 2 | Full | Signal Peptide | No | GI10208 | 2E-084 | XP_002000399 | Drosophila mojavensis |  | **X** |  |  |
| GM-5558  (EZ421936) | [cn9149](http://www.genedb.org/genedb/Search?name=cn9149&organism=glossina) | 1 | Full | Cytoplasmic | Yes | CG12012 | 4E-031 | NP_647813 | Drosophila melanogaster |  | **X** |  |  |
| GM-5560  (EZ421937) | [cn9149](http://www.genedb.org/genedb/Search?name=cn9149&organism=glossina) | 1 | Full | Cytoplasmic | Yes | CG12012 | 4E-031 | NP_647813 | Drosophila melanogaster |  | **X** |  |  |
| GM-5920  (EZ421938) | [cn8858](http://www.genedb.org/genedb/Search?name=cn8858&organism=glossina) | 4 | Full | Cytoplasmic | No | GH17720 | 2E-071 | XP_001994054 | Drosophila grimshawi |  | **X** |  |  |
| GM-1331  (EZ421923) | [FN185196](http://www.ncbi.nlm.nih.gov/nucest/224508616?rid=dc7dkjmy014&blast_rank=1&dopt=genbank&log$=nucltop)* | 27 | Full | Signal Peptide | No | [hypothetical protein](../../../../../Aksoy%20Lab%20-%20Geoffs%20Files/AppData/Local/Microsoft/Windows/Temporary%20Internet%20Files/Content.Outlook/WJGOY5OV/links/NR/GM-1331-NR.txt) | 5E-020 | XP_654805 | Entamoeba histolytica HM-1:IMSS |  |  | **X** |  |
| GM-4319  (EZ421935) | [FN183030](http://www.ncbi.nlm.nih.gov/nucest/224510238?rid=dc7jxr1t01n&blast_rank=1&dopt=genbank&log$=nucltop)* | 7 | Full | Signal Peptide | Yes | GJ11658 | 2E-082 | XP_002047922 | Drosophila virilis |  |  | **X** |  |
| GM-6935  (EZ421956) | [cn13982](http://www.genedb.org/genedb/Search?name=cn13982&organism=glossina) | 3 | Truncated | Signal Peptide | No | GJ14563 | 1E-050 | XP_002058692 | Drosophila virilis |  |  | **X** |  |
| GM-17340  (EZ421925) | [cn13236](http://www.genedb.org/genedb/Search?name=cn13236&organism=glossina) | 1 | Full | Signal Peptide | Yes | GK13359 | 4E-045 | XP_002073059 | Drosophila willistoni |  |  |  | **X** |
| GM-9082  (EZ421954) | [FN185432](http://www.ncbi.nlm.nih.gov/nucest/224507956?rid=dc7smn4g01s&blast_rank=1&dopt=genbank&log$=nucltop)* | 2 | Full | Signal Peptide | No | GA13088 | 8E-031 | XP_001353516 | Drosophila pseudoobscura |  | **X** | **X** |  |
| GM-9034  (EZ421961) | [FN182544](http://www.ncbi.nlm.nih.gov/nucest/224508680?rid=dc7xhgkf01s&blast_rank=1&dopt=genbank&log$=nucltop)* | 2 | Truncated | Undetermined | No | GK12485 | 4E-060 | XP_002067881 | Drosophila willistoni |  |  | **X** | **X** |
| **Unknown Function** | | | | | | | | | | Carcass | Reproductive Tract | Larva | Pupa |
| GM-9042  (EZ421944) | [cn8914](http://www.genedb.org/genedb/Search?name=cn8914&organism=glossina) | 2 | Full | Cytoplasmic | No | IP09048p | 8E-005 | AAY51519 | Drosophila melanogaster |  | **X** |  |  |
| GM-17588  (EZ421929) | [FN184353](http://www.ncbi.nlm.nih.gov/nucest/224509453?rid=dc83zf6n016&blast_rank=1&dopt=genbank&log$=nucltop)* | 1 | Full | Cytoplasmic | No | cell division protein FtsY | 0.21 | ZP_01090162 | Blastopirellula marina |  | **X** |  |  |
| GM-17580  (EZ421927) | [FN184436](http://www.ncbi.nlm.nih.gov/nucest/224510412?rid=dc88657r014&blast_rank=1&dopt=genbank&log$=nucltop)* | 1 | Full | Cytoplasmic | No | GA28476 | 0.062 | XP_002133979 | Drosophila pseudoobscura |  |  | **X** | **X** |
| GM-355  (EZ422129) | [cn14496](http://www.genedb.org/genedb/Search?name=cn14496&organism=glossina) | 10 | Truncated | Undetermined | No | Calcium-binding protein | 5E-033 | XP_001891729 | Brugia malayi |  |  | **X** | **X** |
| GM-9052  (EZ421947) | [FN183535](http://www.ncbi.nlm.nih.gov/nucest/224508915?rid=dc8cm1ht01s&blast_rank=1&dopt=genbank&log$=nucltop)* | 2 | Full | Cytoplasmic | No | leucine-rich repeat family protein / extensin family protein | 2E-010 | NP_193070 | Arabidopsis thaliana | **No Signal** | | | |
| GM-9054  (EZ421971) | [FN184047](http://www.ncbi.nlm.nih.gov/nucest/224510070?rid=dc8khxy801n&blast_rank=1&dopt=genbank&log$=nucltop)* | 2 | Fragment | Undetermined | No | hypothetical protein | 3E-015 | XP_001054508 | Rattus norvegicus | **No Signal** | | | |
| GM-17587  (EZ421928) | [cn2847](http://www.genedb.org/genedb/Search?organism=glossina&name=cn2847&isid=true) | 1 | Full | Cytoplasmic | No | GA10706 | 2E-004 | XP_001354000 | Drosophila pseudoobscura | **No Signal** | | | |
