# Supplementary material for: Transcriptome analysis of reproductive tissue and intrauterine developmental stages of the tsetse fly (Glossina morsitans morsitans)
Source: BMC Genomics. 2010 Mar 9;11:160. doi: 10.1186/1471-2164-11-160 (PMC2846916; doi:10.1186/1471-2164-11-160)
Supplement: Additional file 3 — Primer list supplement. This file contains a table of the primers used in the tissue specificity RT-PCR analysis from table 2. [file 1471-2164-11-160-S3.DOC]

| Database ID (GenBank Accession #) | Primer Sequence |
| --- | --- |
| GM-355 F (EZ422129) | TCTGCATAGCTACCTTGCTGTC |
| GM-355 R (EZ422129) | ACCAGGGTCCTTTAGCTGGT |
| GM-1331 F (EZ421923) | CGACGACGATAGCGATGATA |
| GM-1331 R (EZ421923) | TTCAACACAGCCTGCACTTC |
| GM-4319 F (EZ421935) | GGCAGAGGAAGTTGAAGTGC |
| GM-4319 R (EZ421935) | TGGTATACGGGCAGGTGATT |
| GM-5558 F (EZ421936) | GAACCACCATCAATGGCTTT |
| GM-5558 R (EZ421936) | ATTCGACAAGCTGGACAACC |
| GM-5560 F (EZ421937) | GAACCACCATCAATGGCTTT |
| GM-5560 R (EZ421937) | ATTCGACAAGCTGGACAACC |
| GM-5920 F (EZ421938) | ACGATGTTGTGAAGCTGTGC |
| GM-5920 R (EZ421938) | GCCGCATAAGTTCTTGCATT |
| GM-6933 F (EZ421939) | GGGATGTCGATCAGTCACCT |
| GM-6933 R (EZ421939) | GGTGGCAGGTATTCTTTGGA |
| GM-6935 F (EZ421956) | GTGGCCTGTCTTCAGGTGTT |
| GM-6935 R (EZ421956) | TGATTTTGCGAGATTTGCAG |
| GM-9042 F (EZ421944) | TATGGCTACCAACACCAGCA |
| GM-9042 R (EZ421944) | CGTTGGGTTGCAATAGATGA |
| GM-9082 F (EZ421954) | TGCCCCTGATACGGAAATAG |
| GM-9082 R (EZ421954) | CCTCTGTGGGAAACGTTGAT |
| GM-17340 F (EZ421925) | GCCTTTGTGGTCGGTGTTAT |
| GM-17340 R (EZ421925) | TATAGGCAGTAGCCGCATGA |
| GM-17580 F (EZ421927) | CATGCGGTCGAACAAGACTA |
| GM-17580 R (EZ421927) | ATGTCATTGTGTCCGTTCCA |
| Gm-9054 F (EZ421971) | CAAAGGAAGCTGCAATCACA |
| Gm-9054 R (EZ421971) | TCCTGTTGCTTTTGCTGTTG |
| Gm-9052 F (EZ421947) | AATACTGTCCACATCCAAGG |
| Gm-9052 R (EZ421947) | GCGGCAGTGGATAATATAGA |
| Gm-9034 F (EZ421961) | ATGTCGCTCTCGCTGGTACT |
| Gm-9034 R (EZ421961) | GGTGATGGTGAACGGCTACT |
| Gm-9063 F (EZ421950) | TACTGTGAACGAGGGGAACC |
| Gm-9063 R (EZ421950) | GATCGACCGGACAAACCTAA |
| Gm-17588 F (EZ421929) | CGCAGAGTTGCACCTTATCA |
| Gm-17588 R (EZ421929) | ACGGCGCCTGTCATATCTAC |
| Gm-17587 F (EZ421928) | GGGGTTTCAACCAAATCCTT |
| Gm-17587 R (EZ421928) | AGCGTTGATTTTCAGGGGTA |
